# High-Temperature Tolerance Protein Engineering through Deep Evolution

**DOI:** 10.34133/bdr.0031

**Published:** 2024-04-03

**Authors:** Huanyu Chu, Zhenyang Tian, Lingling Hu, Hejian Zhang, Hong Chang, Jie Bai, Dingyu Liu, Lina Lu, Jian Cheng, Huifeng Jiang

**Affiliations:** ^1^ Key Laboratory of Engineering Biology for Low-carbon Manufacturing, Tianjin Institute of Industrial Biotechnology, Chinese Academy of Sciences, Tianjin 300308, P. R. China.; ^2^ National Center of Technology Innovation for Synthetic Biology, Tianjin 300308, P. R. China.; ^3^ Tianjin Zhonghe Gene Technology Co., LTD, Tianjin 300308, P. R. China.; ^4^ College of Biotechnology, Tianjin University of Science and Technology, Tianjin 300457, P. R. China.

## Abstract

Protein engineering aimed at increasing temperature tolerance through iterative mutagenesis and high-throughput screening is often labor-intensive. Here, we developed a deep evolution (DeepEvo) strategy to engineer protein high-temperature tolerance by generating and selecting functional sequences using deep learning models. Drawing inspiration from the concept of evolution, we constructed a high-temperature tolerance selector based on a protein language model, acting as selective pressure in the high-dimensional latent spaces of protein sequences to enrich those with high-temperature tolerance. Simultaneously, we developed a variant generator using a generative adversarial network to produce protein sequence variants containing the desired function. Afterward, the iterative process involving the generator and selector was executed to accumulate high-temperature tolerance traits. We experimentally tested this approach on the model protein glyceraldehyde 3-phosphate dehydrogenase, obtaining 8 variants with high-temperature tolerance from just 30 generated sequences, achieving a success rate of over 26%, demonstrating the high efficiency of DeepEvo in engineering protein high-temperature tolerance.

## Introduction

Engineering enzymes to enhance their function at high temperature is crucial in multiple fields such as food, feed, biocatalysis, biomedicine, and biomanufacturing [1–3]. At present, directed evolution is the most potent method for improving the thermostability of natural proteins, yet it typically necessitates multiple rounds of random mutagenesis and high-throughput screening [2,4–7], making it labor-intensive and expensive. From an engineering perspective, the theoretical space of possible protein sequences is too large to explore exhaustively, either experimentally or computationally, and the functional proteins within the entire protein sequence space are extremely scarce, making it challenging to identify highly functional sequences among the vast nonfunctional sequence space [8–10]. To address this issue, many rational or semirational strategies [11–14] have been developed to improve the possibility of each mutant possessing the desired function. Moreover, many high-throughput approaches [6,7] have been introduced to increase the rate of experimental screening. Nonetheless, there remains large room for improvement in the efficiency of these tools [15].

Recently, novel deep learning models have been developed to address various biological challenges, such as predicting protein structure [16–18], Enzyme Commission number [19], enzyme turnover [20], gene function [21,22], and also the thermostability of proteins [23]. Although deep learning techniques have achieved notable progress in modifying protein thermostability, the quantity and quality of training data still play pivotal roles in limiting these models’ effectiveness [24]. From a data perspective, procuring data annotated with organisms’ optimal growth temperature (OGT) labels is relatively more straightforward compared to obtaining highly precise protein thermostability property labels. Several OGT-based methods have been developed to identify thermostable proteins [25–27]. However, the relatively ambiguous nature of these OGT data labels, which do not demonstrate a one-to-one correspondence with protein properties, limits the accuracy of these methods. More effective methodologies for harnessing these abundant OGT data are needed [28].

Studies on protein sequence design have demonstrated that deep learning models can learn the diversity of natural protein sequences, enabling the generation of protein variants to expand the functional protein space [21,22,29,30]. In addition, general protein language models, such as UniRep [31] and the Evolutionary Scale Modeling (ESM) [32], can embed the protein sequences into a high-dimensional representation space, in which it is more feasible to establish connections between protein properties and sequence variants [33–35]. These advancements present an opportunity to engineer proteins with high-temperature tolerance by combining 2 deep learning models from an iterative evolution perspective, whereby a generative model produces abundant variants from a reasonable sequence space with the desired function, after which a selective model is used to identify variants with high-temperature tolerance. Theoretically, this idea circumvents the issue of exploring an immense protein sequence space by sampling within a confined functional space with potential high-temperature tolerance traits, and it alleviates the need for high-precision thermostability calculations, which are difficult from typical OGT data, by iteratively obtaining desired variants through manageable selection pressure.

**Fig. 1.**
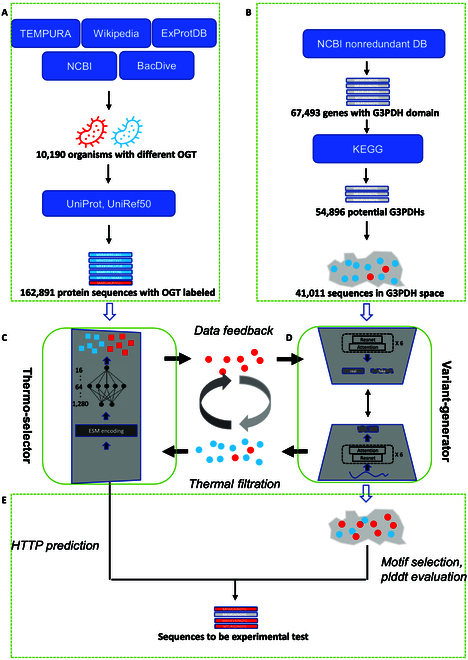
Framework and data flow of DeepEvo. (A) The data collection procedure of sequences from different OGT organisms. (B) The collection of natural sequences with desired function (G3PDH as example). (C) The OGT-labeled sequences were used to train the Thermo-selector. (D) The sequences with desired function were used to train the Variant-generator. The Thermo-selector model was trained and used as a sequence filter to classify HTTP sequences (denoted as red squares) and LTTP sequences (denoted as blue squares), and the Variant-generator was trained to generate reasonable sequences (denoted as circles) in the confined functional protein sequence space, and then the generated sequences by Variant-generator were passed to the Thermo-selector, and the sequences predicted as HTTP (denoted as red circles) were used to refine the Variant-generator by a data feedback procedure. The generation, high-temperature tolerance traits filtration, feedback, and regeneration constructed an iteration procedure to generate suitable sequences with high-temperature tolerance directly. (E) The generated sequences that predicted as HTTP and had suitable functional conservation motif and good structural predictions were selected for experimental verification.

In contrast to a typical directed evolution strategy based on highly labor-intensive iterative mutagenesis, here, we proposed a deep evolution (DeepEvo) strategy to improve protein high-temperature tolerance through functional sequence generation and selection (Fig. 1). Firstly, we leveraged a successful protein language model (ESM) to extract high-temperature tolerance related information from more than 190,000 protein sequences across a wide range of organisms with different OGT and constructed a high-temperature tolerance selection model (Thermo-selector). At the same time, a modified generative model (Variant-generator) based on ProteinGAN was constructed to generate functional sequences. Finally, after iterative optimization of Variant-generator by the output of Thermo-selector, we evaluated the efficiency of DeepEvo for protein high-temperature tolerance engineering on the model enzyme glyceraldehyde 3-phosphate dehydrogenase (G3PDH), which is a key enzyme for glycolysis with important applications in industry and medicine [36,37] (Fig. S1). Through experimental evaluation, 8 high-temperature-tolerant G3PDH variants were obtained from just 30 tested sequences, highlighting the efficiency of the DeepEvo approach in engineering protein with high-temperature tolerance.

## Materials and Methods

### Collection of organisms with different OGTs

We collected organisms with different OGT (mainly microorganism) from 5 sources. The first source is TEMPURA, which is a database of growth temperatures of usual and rare prokaryotes (http://togodb.org/db/tempura) [38]. In the database, we obtained about 8,000 organisms and their OGT data. The second source is ExProtDB [39], which is a database collecting extremophilic proteins and their host organisms; about 300 thermophiles were collected from this database. The third source was Wikipedia web search. We fetched the names of all genome sequenced microorganisms from National Center for Biotechnology Information (NCBI) and then searched the name in the web. If the web contains some key words like “extremophile”, “thermophilc”, “thermophile”, “thermophilic”, “high temperatures”, “thermoacidophilic”, or “polyextremophile”, we check whether the microorganism in the web is a thermophile organism, and about 500 thermophiles were collected this way. The last source is BacDive [40], which represents a collection of organism-linked information covering the multifarious aspects of bacterial and archaeal biodiversity. We collected about 5,000 microorganisms in the database, which includes the information of growth temperatures. In totally, we collected about 10,190 organisms, some of which without the information of OGT were individually searched in website. Among them, we define 805 organisms (with OGT ≥ 50 °C), 5,122 organisms (with OGT ≥ 30 °C, <50 °C) and 4,262 organisms (with OGT < 30 °C) as high-temperature organisms (HTO), middle-temperature organisms (MTO), and low-temperature organisms (LTO), respectively.

### Collection of genes with different OGT labels

For the collected microorganisms with the information of growth temperatures, we respectively fetched the corresponding genes from 3 downloaded gene sets (i.e., UniProt reference proteomes, UniRef90 and UniRef50). The fetched genes were further divided into HTO, MTO, and LTO genes. In UniProt reference proteomes, we totally obtained 25,724,264 genes, which include 1,393,345 HTO, 12,317,734 MTO, and 12,013,185 LTO genes, respectively. In UniRef90, we totally obtained 15,901,817 genes that include 973,655 HTO, 7,941,331 MTO, and 6,986,831 LTO genes, respectively. In UniRef50, we totally obtained 2,199,998 genes that include 165,625 HTO, 1,120,580 MTO, and 913,793 LTO genes, respectively. These genes were considered as training set for the construction of high-temperature tolerance selection model.

### Collection of G3PDH genes

To construct gene generative model, we screened and analyzed all potential G3PDHs in the NCBI database. First, we predicted all potential G3PDHs by search against the nonredundant database with the Pfam domain ID PF02800 (hmmscan --cpu 10 --domtblout output.txt -E 1e-4 PF02800.hmm NR.fasta), and 67,493 genes with the domain were obtained. Second, we retrieved all G3PDHs from Kyoto Encyclopedia of Genes and Genomes (KEGG) database (https://www.genome.jp/dbget-bin/get_linkdb?-t+genes+pf:PF02800). Third, we made a local blastp search using G3PDHs from NCBI as the query sequences, and G3PDHs from KEGG as the BLAST database. After blastp search, 54,896 potential G3PDHs were screened with 3 standards: the best hit is a G3PDH (Enzyme Commission number:1.2.1.12, KEGG Orthology identifier: K00134), the identity is more than 40, and the align length is more than 200. After filtering too long and too short genes, more than 40,000 potential G3PDHs were selected to construct generative model.

### Building and training the Thermo-selector

To identify sequences of high-temperature-resistant proteins, a 2-part dataset was compiled, consisting of 30,968 high-temperature sequences (OGT ≥ 50 °C) and 162,890 low-temperature sequences (OGT < 30 °C). These sequences are range from 300- to 800-amino-acids length, and the sequence identity between each other are no more than 50%. Seventy percent of the sequences were chosen as training set, and others remained as testing set. The training data was first preprocessed by encoding each sequence into a 1,280-dimensional vector using the pretrained ESM-1b model. These encoded vectors were then used to train a 3-layer multilayer perceptron with dimensions 1,280:64:16 and a binary cross-entropy loss function. An Adam optimizer was used to train the model with a learning rate 1 × 10^−3^. Seventy-five epochs of training were performed to make the loss stable. The model was evaluated using standard metrics such as precision, recall, and F1 score (Supplementary Methods). The pytorch framework was used for building this model.

### Building and training the Variant-generator

To build the Variant-generator, we filtered the collected G3PDH sequences with the length >300 and <800 amino acids. A total of 41,011 sequences were used for training and testing. We randomly split these sequences in the ratio of 9:1 as the training set and test set, respectively. The GAN architecture to generate G3PDH sequences was based on the ProteinGAN model. The discriminator and generator networks were built by ResNet blocks which contained 3 convolution layers with rectified linear unit activations and a transformer block with muti-head attention mechanism. A random vector of 128 values was used as the input to the generator, and the output matrix dimensions were 512 × 21, which was correspond to the one-hot encoded sequence of length 512 with a 21 words vocabulary (20 amino acids and a sign for gaps at the beginning or ending of the sequence). The matrix with the same dimensions as the output of the generator is used as input to the discriminator. In the training process, the generator generated 64 sequences as a batch, and these generated sequences were mixed with 64 natural G3PDH sequences sampled in the training set based on the sampling weights described above, and then they were passed to the discriminator for discrimination. A nonsaturating loss with R1 regularization was used as loss function in this model, and we selected the Adam algorithm for optimizing the networks. The learning rate was gradually decreased from 1 × 10^−3^ to 5 × 10^−5^. The model was trained for 200,000 steps, which took about 12 hours on a Nvidia GTX2080Ti system.

### Analysis of generated sequences

A distance matrix of cluster representatives was used as the t-distributed stochastic neighbor embedding (t-SNE) input. To obtain cluster representatives, the numbers of sequences in natural and generated datasets were first equalized by randomly taking 10,000 sequences from each dataset. These sequences were independently clustered using MMseqs2 with 80% minimal sequence identity. Representative sequences of these clusters were chosen based on the MMseqs2 output. From the representative sequences, a distance matrix was generated using Clustal Omega. The distance matrix was used with the scikit-learn t-SNE module with default settings, except that the embedding generation perplexity was set to 7. Coordinates given by t-SNE were used for plotting and the size of a given dot was visualized based on the cluster size it represents.

### Select generated sequences for experimental verification

To filter out representative sequences for experimental verification, we first used the discriminator part of the GAN-based Variant-generator to score the generated sequences. After ranking the generated sequences by this score, the top 20% that were strongly discriminated as natural-like sequences were kept. We then used a crystallized G3PDH (PDBID: 3KV3) as a template, extracted the nicotinamide adenine dinucleotide (NAD) and 3-phosphoglyceric acid (G3P) binding positions (residue numbers 12, 13, 35, 78, and 316 for NAD binding, and 151, 152, 181, 183, and 234 for G3P binding) to construct a functional motif. We then aligned the generated sequences to the template, and if there was no gap in the functional motif region, the sequences were retained. We then calculated the identities of the generated sequences and the natural sequences with blastp. Tens of sequences were selected with different levels of variation (60% to 90% for the original Variant-generator and 80% to 90% for the refined Variant-generator). The selected sequences were then structurally modeled with Alphafold2, keeping those with predicted local distance difference test (plddt) score >90%.

### Expression and purification of proteins

Protein coding DNA sequences mentioned in this study were all synthesized, cloned into pET28a expression vector between NdeI and XhoI, and then sequence-verified by Zhong He Gene Co. Ltd (Tianjin). The constructs were transformed into BL21 (DE3) or Arctic Express (DE3) *E. coli*. Cells were seeded in 2YT medium (kanamycin, 50 μg/ml) at a ratio of 1:160 and grown at 37 °C, 220 rpm. After OD600 (optical density at 600 nm) of cells were reaching 0.4 to 0.6, isopropyl β-d-1-thiogalactopyranoside was added to a final concentration of 0.5 mM to induce expression. Strain cells were cultured at 16 °C, 220 rpm overnight and then harvested by centrifugation. Cells were resuspended in lysis buffer (50 mM tris-HCl, pH 6.8) and lysed by using a high-pressure homogenizer at 1,200 to 1,500 bar, for 2 to 3 times. Cell debris was discarded by centrifugation at 10,000 × g for 40 min. The Ni-nitrilotriacetic acid agarose column was balanced with double-distilled water and lysis buffer for 2 column volume. The supernatant was applied to the column then proteins were eluted using a gradient of elution buffer (50 mM tris-HCl with 10, 50, and 200 mM imidazole). The fractions were then collected and analyzed by sodium dodecyl sulfate-polyacrylamide gel electrophoresis. Purified proteins were concentrated by centrifugation (4,000 × g, 30 min) in 10-kDa ultrafiltration tubes (Centriplus YM series, Millipore) and flash-frozen in liquid nitrogen then stored at −80 °C.

### G3PDH activity and high-temperature tolerance assay

The assay for G3PDH activity was carried out according to the method originally described by Ferdinand with minor modifications [41]. Briefly, the activity can be monitored by measuring the formation of NADH. Triplicate samples of purified proteins were mixed with 10 mM NAD in 993-μl reaction (40 mM triethanolamine, 50 mM Na_2_HPO_4_, 5 mM EDTA, and 0.1 mM DTT, pH 8.6) separately. G3PDH solution was added into the system, and the A340 was determined immediately. The reaction system was incubated at 30 °C for 10 min and the A340 was determined again. The activity of G3PDHs were calculated with the formula Units = △A340 × VT(Volume of tube)/(6.22 × Concentration(mg) × Time(s)). For the high-temperature tolerance assay, 100-μl reaction systems were developed in 96-well plates. The plates were incubated at designed temperature in a thermostable microplate reader with persistent reading of A340 for 30 min.

### Melting temperature (Tm) analysis

The purified G3PDHs were analyzed for their thermal stability using a differential scanning fluorimetry method with Uncle system (Unchained Labs). Protein samples (0.5 mg/ml) fluorescence was monitored in the temperature range of 20 to 95 °C with 0.8 °C/min heating rate. The fluorescence intensity over the spectrum between 300 and 430 nm were measured and analyzed using UNcle Analysis Software (Unchained Labs) and the Tm values were determined.

## Results

### Construction of the high-temperature tolerance selection model: Thermo-selector

Like natural selection, the DeepEvo approach utilized a high-temperature tolerance selection model to identify protein sequences with potential for enhanced high-temperature tolerance. Since this model serves solely as a source of selection pressure in the virtual evolution process, there is no need to construct a high precision predict model for thermostability relevant metrics. Instead, a binary classification model is sufficient to achieve this goal. As such, the high-temperature tolerance selection model was designed to predict whether a protein is a high-temperature-tolerant protein (HTTP) or low-temperature-tolerant protein (LTTP). Considering that the total proteins of organisms that survive in high-temperature environments should be HTTP, the OGT of the organisms were used as a label to measure the temperature tolerance of the natural protein. To obtain enough labeled data for supervised learning, the information of 10,190 organisms with a wide range of OGT was collected from TEMPURA [38], ExProtDB [39], NCBI, and BacDive [40]. Then, more than 20 million corresponding genes were retrieved from UniProt and UniRef gene sets. Considering the primary objective of our model is to discriminate HTTP sequences, in order to enable the model to learn more pronounced features of temperature tolerance, we exclusively utilized the portion of the OGT data that comprises high OGT and low OGT. This approach was adopted to reduce the ambiguity at the discriminative threshold of the model. The corresponding protein sequences from organisms with OGT > 50 °C or OGT < 30 °C were defined as HTTPs or LTTPs, respectively (Fig. 1A and Materials and Methods). To reduce the impact of sequence similarity on the thermostability-related traits, only sequences with pairwise identity less than 50% were retained. Given the length of most enzymes used in practical applications, proteins with a length >300 and <800 amino acids were retained. After filtering based on these criteria, a total of 30,968 HTTPs and 162,890 LTTPs were collected to build the selection model (Fig. 1 and Table 1).

**Table 1.** Summary of the thermo-selector model

Parameters			
Batch size	100		
Rounds	75		
		Training set	Testing set
Data	HTTPs	21,772	9,246
	LTTPs	113,978	48,912
Metrics	Accuracy	97.8%	95.1%
	Precision	97.0% for HTTPs	86.0% for HTTPs
	Recall	87.7% for HTTPs	78.0% for HTTPs

Inspired by natural language processing techniques, the ESM-1b pretraining model was used to encode the training data as a 1,280-dimensional vector. Using the ESM embedding vectors as input, a 3-layer fully connected neural network was built (Table 1). Using 70% of the collected data as the training set, the model was optimized by cross-entropy loss. After 75 rounds of training, the loss function of the model became stable (Fig. S2). After the training procedure, the overall accuracy of the model on the testing set comprising the remaining 30% of the data was 95.1% (Fig. S3), indicating that most of proteins in the testing set were correctly classified into HTTPs or LTTPs. Considering the imbalance of our training set, with 84% of total sequences belonging to LTTPs, we calculated the precision and recall to further evaluate the performance of our model on the tested HTTPs (Supplementary Methods). These measurements showed that 78.0% (recall) of all labeled HTTPs were predicted as HTTPs by the model, and 86.0% (precision) of all sequences predicted as HTTPs by the model were actually the labeled HTTPs in the test set (Table 1). Unlike state-of-the-art methods in protein thermostability prediction that utilize OGT data, such as DeepET [25] or Tome [26], our model does not directly predict metrics related to thermostability. Instead, it classifies sequences exhibiting HTTP traits, subsequently leveraging this classification as a pression in a virtual evolutionary process. With a simpler target which is to accumulate the potential sequences with HTTP traits, our model is more robust to ambiguous training data, which may limit the accuracy of prediction or regression models. Recently, another OGT-based classification model named as TemStaPro [27] with more complex structure showed similar performance on our test dataset with a 78.7% recall for HTTPs. Considering the evolutionary methodology, these results suggest that our model can be used as a viable filter for identifying HTTPs even though the performance is moderate. This high-temperature tolerance selection model was named Thermo-selector.

### Construction of a variant generation model for G3PDH: Variant-generator

To generate functional sequences more efficiently in a confined sequence space by the DeepEvo approach, a G3PDH Variant-generator was built by revising ProteinGAN with the multiheaded attention mechanism (Fig. S4). This model structure includes a sequence generator and a discriminator. The generator attempts to generate functional sequences and the discriminator attempts to distinguish the generated sequences from the natural sequences. By searching with G3PDH functional domain and filtering with sequences length and identity, 41,011 natural G3PDH-like sequences were extracted from the NCBI, KEGG, and Pfam databases to train the model (Fig. 1B and Materials and Methods). At each training step, starting from a random vector, the generator produced 64 sequences, which were mixed with the same number of natural G3PDH sequence. The discriminator then compared the generated sequences with the natural sequences, which were used to adjust the parameters of both the generator and the discriminator. After 200,000 training rounds, the sequences produced by the generator could not be distinguished clearly from the natural G3PDH sequences by the discriminator (Figs. S5 and S6).

**Fig. 2.**
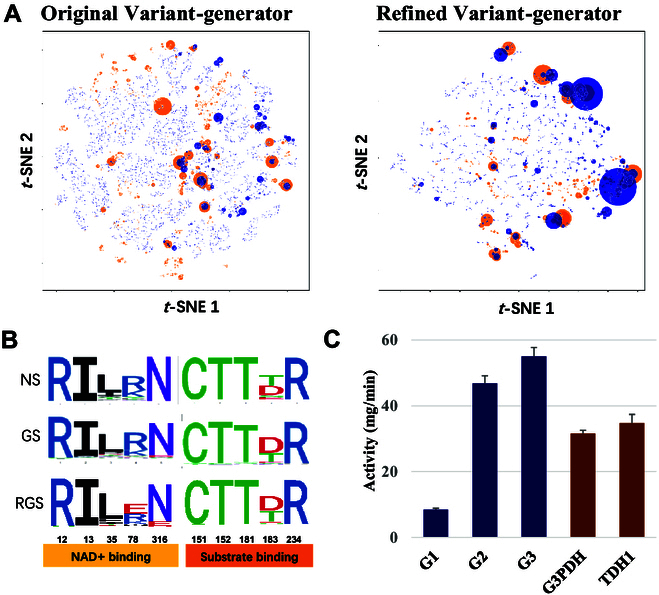
Evaluation of the sequences generated by Variant-generator. (A) t-SNE maps of generated sequences by original Variant-generator and refined Variant-generator. Sequences were classed in the 2-dimentional t-SNE space. The natural sequences clusters are shown as orange circles and the generated sequences clusters are shown as blue circles. The area of the circles indicates the size of the clusters. (B) Sequence logos of binding pockets of natural sequences (NS), original generated sequences (GS) and refined generated sequences (RGS). The conserved positions are grouped in NAD+ and substrate binding. (C) The activity of G3PDHs generated by the original Variant-generator at 30 °C. G1, G2 and G3 represent the 3 generated sequences, a commercial G3PDH from rabbit and the G3PDH from yeast (TDH1) were used as control.

To evaluate the quality of these generated sequences, we conducted t-SNE dimensionality reduction on the natural and generated G3PDH sequences (Fig. 2A, left panel). The generated sequences covered a similar distribution to that of the natural sequences and were grouped into smaller clusters and interpolated within the natural sequence clusters, indicating that the Variant-generator model expanded the functional sequence space of natural G3PDHs. To verify the evolutionary properties reflected in the statistics of amino acid variation, we computed Shannon entropies for each position in multiple sequence alignments of the generated and natural G3PDH sequences. The positional variability of the generated sequences was highly similar to that of the natural sequences (Fig. S7). We also evaluated the highly conserved regions related to the function of G3PDH and found that the generated sequences captured these key positions faithfully (Fig. 2B).

To further evaluate the function of the generated sequences, we sorted them based on the score of the discriminator and filtered them based on the key functional conserved sequence motifs (Materials and Methods). Then, 10 sequences with different similarities to the natural sequences were selected as input for AlphaFold2 to build protein structure, and 6 sequences (G1 to G6) with high plddt scores (>90%) were selected for further experimental validation. Among the 6 proteins, 3 (i.e., G1, G2, G3) not only folded correctly in *E. coli* expression systems (Fig. S8A) but also displayed normal G3PDH activity in in vitro (Fig. 2C). G2 and G3 even showed higher activities than the natural G3PDH from yeast and a commercial G3PDH from rabbits. However, when tested at 65 °C, these 3 generated G3PDH variants lost enzyme activity (Table S1). These experiments proved that the Variant-generator could efficiently generate functional variants from the confined enzyme sequence space. As expected, the directly generated sequences exhibited minimal tolerance to high temperatures since no explicit optimization toward this target.

### Development of the DeepEvo process

Based on the good performance of Thermo-selector and Variant-generator, we further implemented the DeepEvo approach by iterating the 2 models to enhance sampling in the G3PDH functional sequence space for variants with enhanced high-temperature tolerance (Fig. 1C and D and Fig. S4). Benefiting from the GAN structure of the Variant-generator, higher quality generative sequences can be obtained directly from the discriminant scores of the discriminator. 18,238 sequences were selected from the 100,000 generated sequences of the initial Variant-generator based on the discriminator score and the functional conserved residues of G3PDH. Then, the selected sequences were input into the Thermo-selector, where only 1,354 (7.4%) variants were classified as HTTPs. Finally, these 1,354 HTTPs were mixed with all natural HTTPs and added back to the training set of the Variant-generator to refine the model. When the Variant-generator stabilized again, we obtained a refined Variant-generator, which displayed a better HTTP generation performance, as the proportion of HTTPs among the generated sequences increased to 14.9%. Additionally, using the discriminator score as metric, we observed that the sequences generated by the refined Variant-generator were largely consistent with those of the initial variant-generator (Fig. S6). Interestingly, the t-SNE analysis of the newly generated sequences yielded some bigger clusters, suggesting that the generated sequences were enriched in sequence spaces, which is similar to gene family evolution in nature. This indicated that the iterative process of DeepEvo might recapitulate certain unsought mechanisms of the natural evolution process (Fig. 2A, right panel).

**Fig. 3.**
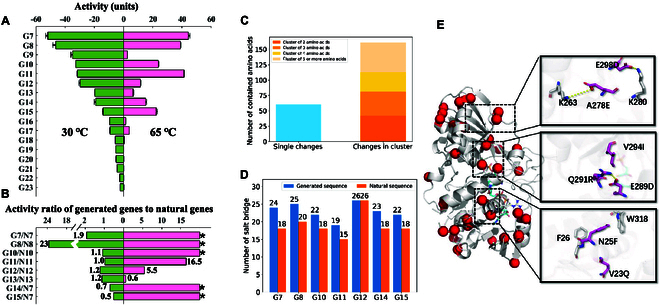
Evaluation of the high-temperature tolerance G3PDH generated through DeepEvo. (A) Catalytic activities of 17 generated G3PDHs at 30 and 65 °C, respectively. The activity unit is defined in Materials and Methods. (B) Activity ratio of 8 verified HTTPs to their individual nearest natural sequence. “*” refers to no activity was detected in natural sequence, and the denominator is 0 in ratio value calculation. (C) Histogram of single changes and clustered changes of mutations in 7 high-temperature activity substantially improved variants. Clusters contains different number of amino acids are shown in different color. (D) Salt bridge numbers of verified HTTPs compared with the number of there corresponding natural proteins. The numbers are calculated by ESBRI (*42*). (E) Structure model of G8 and the single point mutations (red dot) compared to N8. Substrate and coenzyme are shown as blue sticks. Three clusters of mutations are shown in right black boxes.

To further evaluate the high-temperature tolerance of the sequences generated by the refined Variant-generator, 30 sequences (G7-G36) were selected from 2,760 newly generated HTTPs for experimental validation based on the discriminator score, conserved residues, similarity to the nearest natural sequences and AlphaFold2 plddt scores (Fig. 1E and Materials and Methods). These sequences exhibited an average 61% sequence identity among themselves (Fig. S9), and a range of identities (~70 to ~90%) to their nearest natural sequences in the training set (Table S2). The 30 selected sequences were synthesized and then expressed in *E. coli* for protein purification. Among the 30 proteins 23 (77%) were soluble and could be purified (Fig. S8B to D), 17 of which (57%) showed normal G3PDH catalytic activity at 30 °C in the subsequent G3PDH activity assay (Fig. 3A and Table S2). We found that 11 out of the 17 proteins showed detectable activity at 65 °C, with 8 of them (i.e., G7, G8, G10, G11, G12, G13, G14, and G15) exhibiting relatively high-temperature tolerance (Fig. 3A and Materials and Methods), even retaining activity at 70 and 75 °C (Fig. S10). Notably, the nearest natural homologs of 7 among the 8 proteins showed low or even no detectable activity at 65 °C (Fig. 3B), even one of them (N12) was from a high-temperature organism, indicating that the DeepEvo approach indeed can effectively engineer natural LTTPs into HTTPs, rather than merely identifying sequences with high identities to natural HTTPs.

Besides measuring the enzyme activity at high temperatures, we also measured the Tm values of the G3PDH variants with high-temperature tolerance (Table 2). Compared with the G3PDH control from *E. coli* with a Tm value of 50.5 °C, these variants had a substantial increase of 10 to 30 °C in their Tm values, indicating that they had better thermal stability. We also measured the Tm values of the nearest natural homologs of these variants, and the data showed that the generated sequences we selected had better thermal stability than their corresponding nearest natural homologs, which again indicated that the DeepEvo approach had the ability to engineer LTTPs into HTTPs.

**Table 2.** Tm values of generated G3PDHs with high-temperature tolerance and their corresponding nearest natural homologs

Variants	Tm (°C)	Tm of nearest natural homologs (°C)
G3PDH from *E. coli*	50.5 ± 0.4	-
G7	77.8 ± 0.7	54.8 ± 2.6
G8	66.8 ± 1.0	58.7 ± 1.6
G10	60.6 ± 1.0	58.2 ± 0.1
G11	60.8 ± 1.3	58.0 ± 1.8
G12	65.5 ± 0.4	58.9 ± 1.2
G13	66.9 ± 0.9	58.0 ± 1.5
G14	81.9 ± 3.7	54.8 ± 2.6
G15	80.9 ± 5.7	54.8 ± 2.6

To demonstrate the effect of the iteration procedure, we selected 30 sequences from the 1,354 variants generated in the first round with same criteria. Eight of these demonstrated normal G3PDH catalytic activity at 30 °C, and 4 of them showed detectable activity at 65 °C (Fig. S14). In addition to G3PDH, we also did one round of generation and Thermo-selector filter of malate dehydrogenase, which has been used for the evaluation of multiple protein language models [21,22]. Five of 20 experimental tested variants exhibited activity at 65 °C (Fig. S15). These results align with the moderate performance of the Thermo-Selector, which could find some high-temperature tolerance variants, but the overall experimental success rate was unsatisfactory. The iteration procedure for G3PDH improved the ratio of experimentally detected variants with activity at 65 °C from 4 out of 8 to 11 out of 17. The proportion of soluble variants with G3PDH activity and high-temperature tolerance increased with the iterative process, suggesting that both the intrinsic functional properties of the G3PDH and the high-temperature tolerance traits are accumulated during the iteration. The malate dehydrogenase results also exhibited the general applicability of Thermo-selector, highlighting the potential for broader application of our DeepEvo approach.

### Deciphering the design art of DeepEvo 

In order to comprehensively understand the design art of DeepEvo, we compared 7 generated HTTPs having been substantially improved activities at 65 °C with their nearest natural LTTPs, finding that the natural LTTPs require the mutation of approximately 20 to 50 residues to become our generated HTTPs (Table S2). We calculated the predicted ddG of these mutations by DeepDDG and found that no mutations that substantially boost folding free energy. We observed many alanine to serine mutations, (Fig. S12) which may increase the coordination of local hydrogen bonding networks. In addition, we found that a high proportion of mutations introduced charged residues, resulting in a substantial increase in the number of salt bridges [42] in most of the verified HTTPs (Fig. 3D), which could strengthen the local residual interactions and may be a major reason for the stability of the generated HTTPs [43]. The remaining mutations mainly introduced the same type of amino acid (Fig. S12) and generally did not have particularly strong effects on the local side-chain arrangements. Structural analysis showed that the mutated amino acid resides were mainly distributed on the protein surface, with only a few occurring near the catalytic pocket (Fig. S11). Interestingly, we found that two-thirds of the point mutations formed spatial clusters or mutation networks, in which mutants with at least 1 backbone alpha carbon atom (CA) are within 8Å of the CA of other mutants. Conversely, only one-third of the point mutations were single changes (Fig. 3C and Table S3). These results suggest that the DeepEvo approach can enhance local structural interactions and compensate for the deleterious effects of single point mutations through the interaction of multiple mutation sites, which is a huge challenge for the conventional directed evolutionary approaches.

In order to scrutinize the interaction of spatial clusters, we compared the protein G8 with its nearest natural sequence N8, since it showed a great increase of both enzyme activity and thermal stability (Fig. 3A and B). We observed a total of 34 residue changes and 25% more electrostatic interaction pairs in G8 than in N8 (Fig. 3D), which may contribute the overall improved stability of G8 at high temperature according to MD simulations (Fig. S13). Among these mutations, approximately 65% (22/34) were located in 7 spatial clusters (Fig. 3E and Table S3). For example, the A278E mutation in cluster 1 added a new salt bridge, which could change the local position of the adjacent K280. In order to keep the original salt bridge with K280, DeepEvo made the additional mutation E298D to compensate for the distance change (Fig. 3E, top). Similar to cluster 1, the mutation Q291R in cluster 2 would add a pair of salt bridges, but 2 extra mutations (E289D and V294I) occurred nearby, compensating for the effect of changed residue volume (Fig. 3E, middle). Different from clusters 1 and 2, an enhancement in local hydrophobic stacking was observed in cluster 3, in which a π-π interaction was added to strengthen the interaction between the helix bundle through N25F and a nearby residues, while a V23Q mutation might compensate for the increased solvent exposure in the opposite direction (Fig. 3E, bottom). These results indicate that the DeepEvo approach did not simply increase local interactions but also changed the surrounding residues in clusters to achieve a more reasonable local structure, which is often a challenge for conventional enzyme engineering [44,45]. Thus, the DeepEvo approach, using the Variant-generator to consider the context of residues, may enable much deeper sampling in the confined functional sequence space. This new design art, which relies on the synergistic action of multiple mutant sites, may be useful in overcoming local optima.

## Discussion

**Fig. 4.**
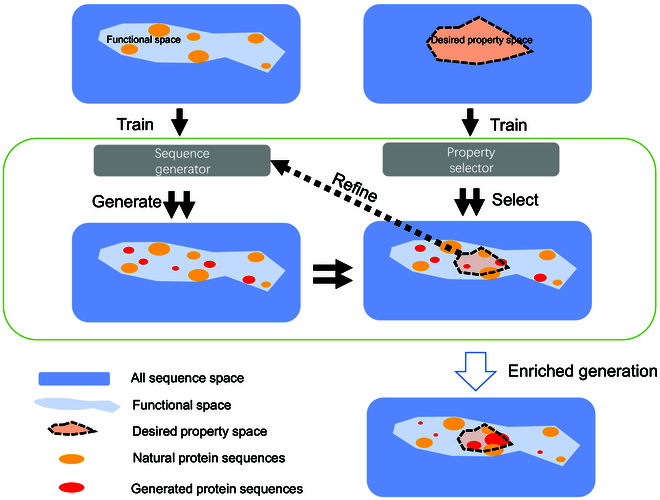
DeepEvo in the perspective of sequence space. In the vast full sequence space of proteins, the functional sequence space occupies only a small fraction. Natural sequences cluster like islands in the functional space (yellow circles). The DeepEvo strategy can sample much deeper into the functional space using a trainable generator that fills in the gaps between natural sequence islands (red circles). For desired properties, we can train special property selectors to filter the generated sequence into the desired property space and refine the sequence generator. After iterations, the sequences sampled by DeepEvo can be enriched in the desired property space, improving the efficiency of obtaining proteins with desired properties.

In this study, we have demonstrated DeepEvo as a highly efficient approach for engineering enzyme with high-temperature tolerance. By combining a thermostability selection model (Thermo-selector) with a functional sequence generator (Variant-generator) in an iterative evolutionary paradigm, DeepEvo achieved over 26% success in generating high-temperature tolerance G3PDH variants. Experimental validation verified 8 highly stable candidates with preserved enzymatic activity out of just 30 tested DeepEvo-designed sequences. Like natural and directed evolution [46,47], the iteration procedure played a fundamental role to the efficiency of the DeepEvo approach. The iteration procedure, which uses the generated HTTPs screened by the Thermo-selector to refine Variant-generator, accumulates thermostable traits in a process similar to natural evolution [48]. Given the acceptable experimental success rate and the inherent limitations of the generative adversarial network (GAN)-based generation model, which cannot guarantee a very high proportion of well-performing proteins, we did not add further rounds of iteration. Compared to previous directed evolution studies usually requiring the screening of at least thousands of mutant clones [2,47,49], from the perspective of sampling the protein sequence space, we have demonstrated the concept of DeepEvo yielded order-of-magnitude improvements in efficiency for obtaining typically enzymes with desired property, which may increase the likelihood of generating desired property relevant sequences, thereby improving the screening efficiency (Fig. 4).

Current data-driven methodologies in the domain of protein thermostability prediction predominantly concentrate on predicting the impact of mutations by metrics change such as melting temperatures or binding free energies [50,51]. Nevertheless, the scarcity of high-quality, unbiased training data for these specific attributes has impeded the performance of predictive models. While several protein stability databases facilitate machine learning predictor development [52,53], progress over the past decade has been modest [28]. This suggests that innovative approaches to data utilization and model development are imperative. In contrast with conventional stability prediction methods relying on scarce high-resolution data, our technique leverages large amount of OGT data from genomic sequences across diverse organisms. Some prior efforts have utilized OGT data to achieve moderate protein thermostability discrimination [25,27]. For instance, TemStaPro [27] exhibits higher accuracy and recall than our Thermo-selector. However, intrinsic ambiguities in OGT data limit applications for guiding engineering stable variants. Proteins from high-OGT organisms tend to have enhanced resilience, yet many sequences from lower OGT species retain stability. This imbalance reduces prediction fidelity experimentally. When testing the 17 soluble G3PDH we generated, less than one-third were accurately predicted by TemStaPro based on our experimental results (Table S2). Rather than focusing on precisely predicting, our Thermo-selector extracts potential high-temperature tolerance traits from OGT data as an evolutionary selective pressure. This makes the iterative DeepEvo process robust to errors from mislabeling truly thermostable sequences that arise from lower-OGT organisms. By innovatively integrating OGT-supervised models with modern generative architectures, DeepEvo provides a new approach for efficiently leveraging ever-expanding genomic data resources to engineer finely tuned protein properties.

In summary, the DeepEvo approach employs an iteration process consisting of generation and selection to effectively produce protein sequences that possess strong foldability and high-temperature tolerance. In the future, it is possible to apply the DeepEvo approach for engineering other protein properties such as acid–base tolerance, catalytic activity, and antigen affinity, allowing for the generation of new proteins with diverse desired properties. Since the performance of generation model importantly impact the output of DeepEvo and the current GAN-based model make us must train Varint-generator individually for the target proteins, we aim to explore the integration of generative frameworks from the fields of natural language processing and image processing to enhance the sequence generation results and general applicability. This will further expand the potential of protein engineering through our DeepEvo approach.

## Data Availability

The training data for the Variant-generator and Thermo-selector in our DeepEvo approach and the parameters of a working Thermo-selector are provided along with a sample usage script at https://zenodo.org/records/8015819. A web server that can utilize the Thermo-selector can be found at https://enzymepred.biodesign.ac.cn.
