# Supplementary figures and images for: High-Temperature Tolerance Protein Engineering through Deep Evolution
Source: Biodes Res. 2024 Apr 3;6:0031. doi: 10.34133/bdr.0031 (PMC10988389; doi:10.34133/bdr.0031)

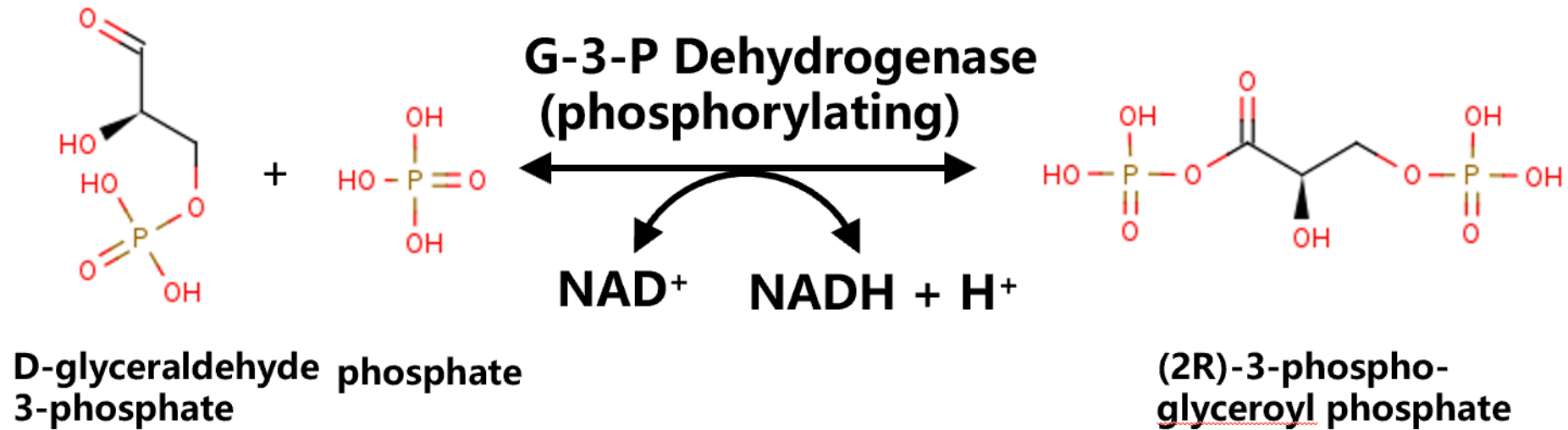

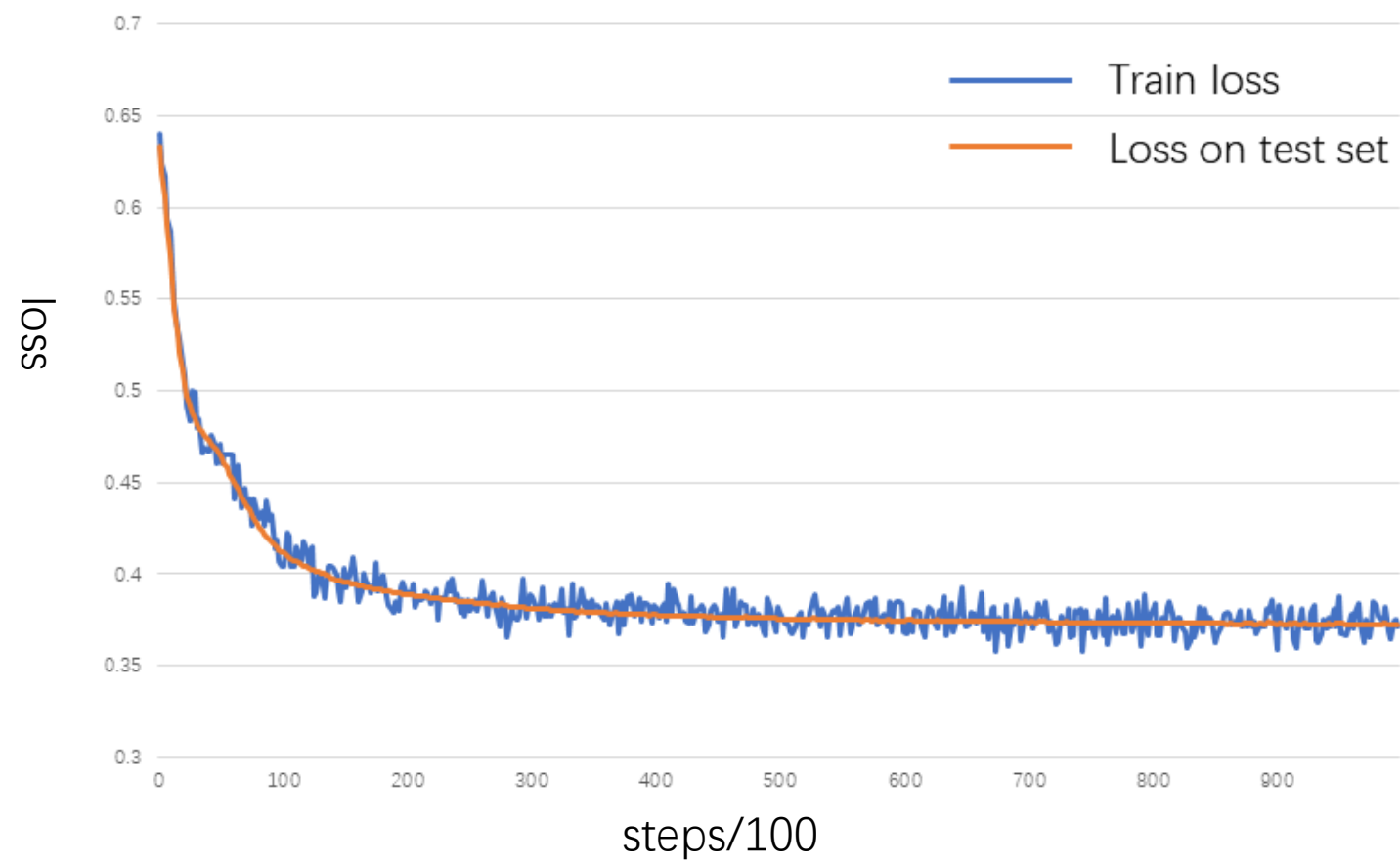

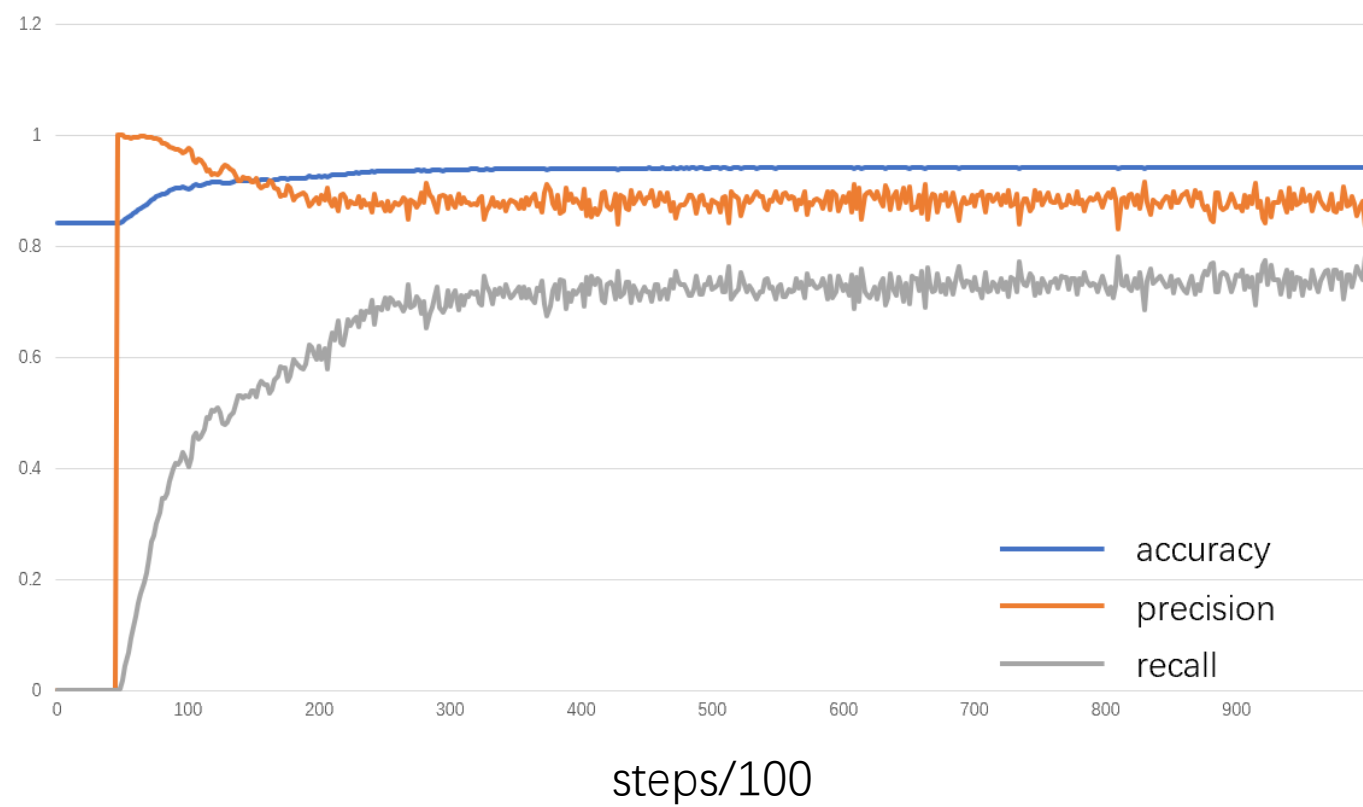

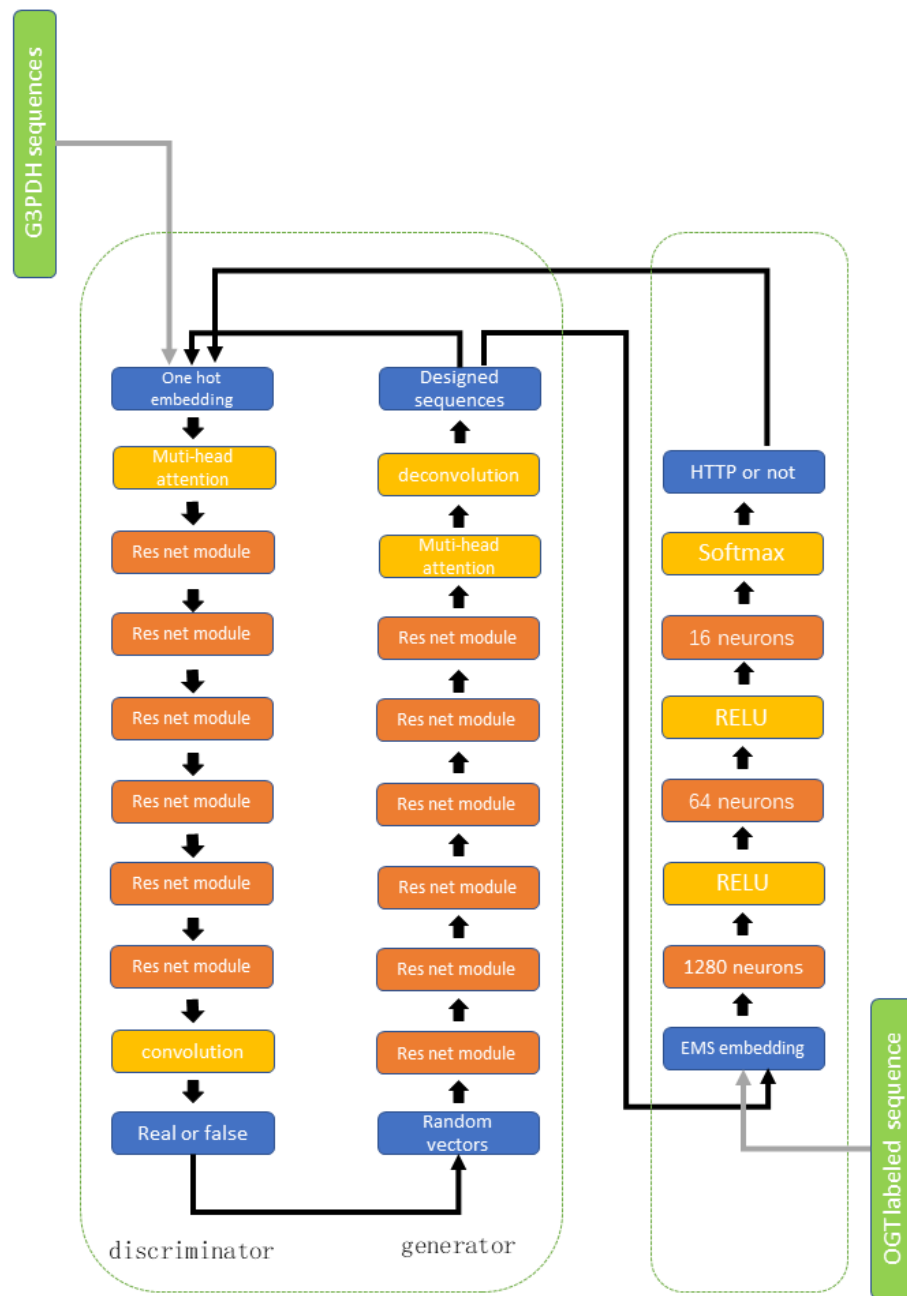

Variant-generator

Thermo-selector

(A)

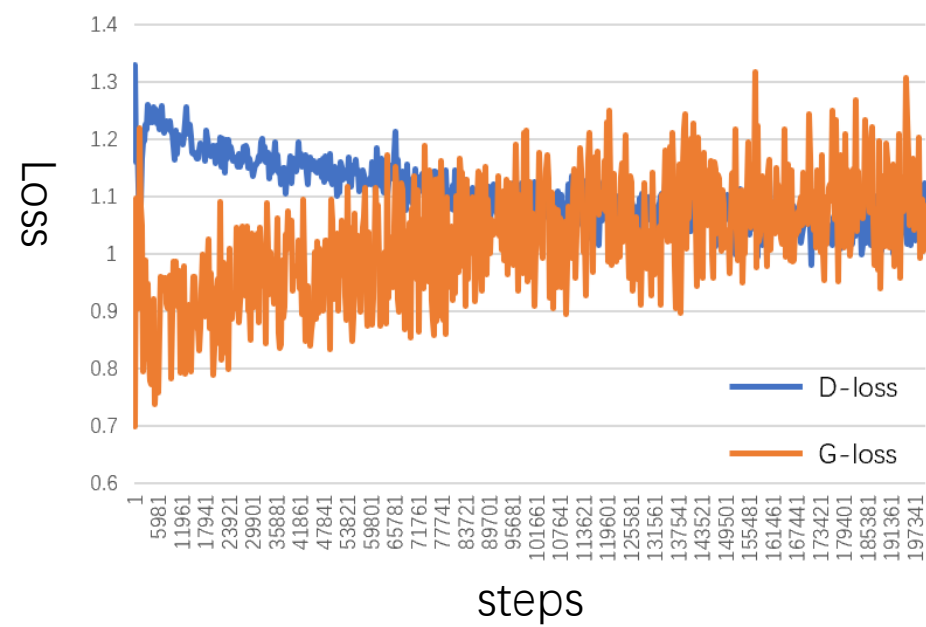

(B)

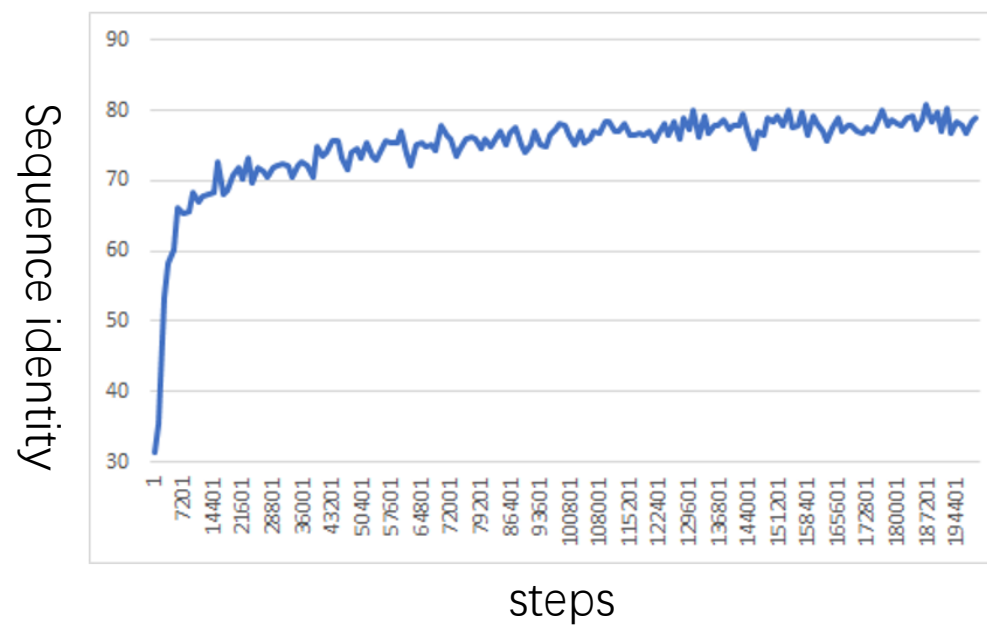

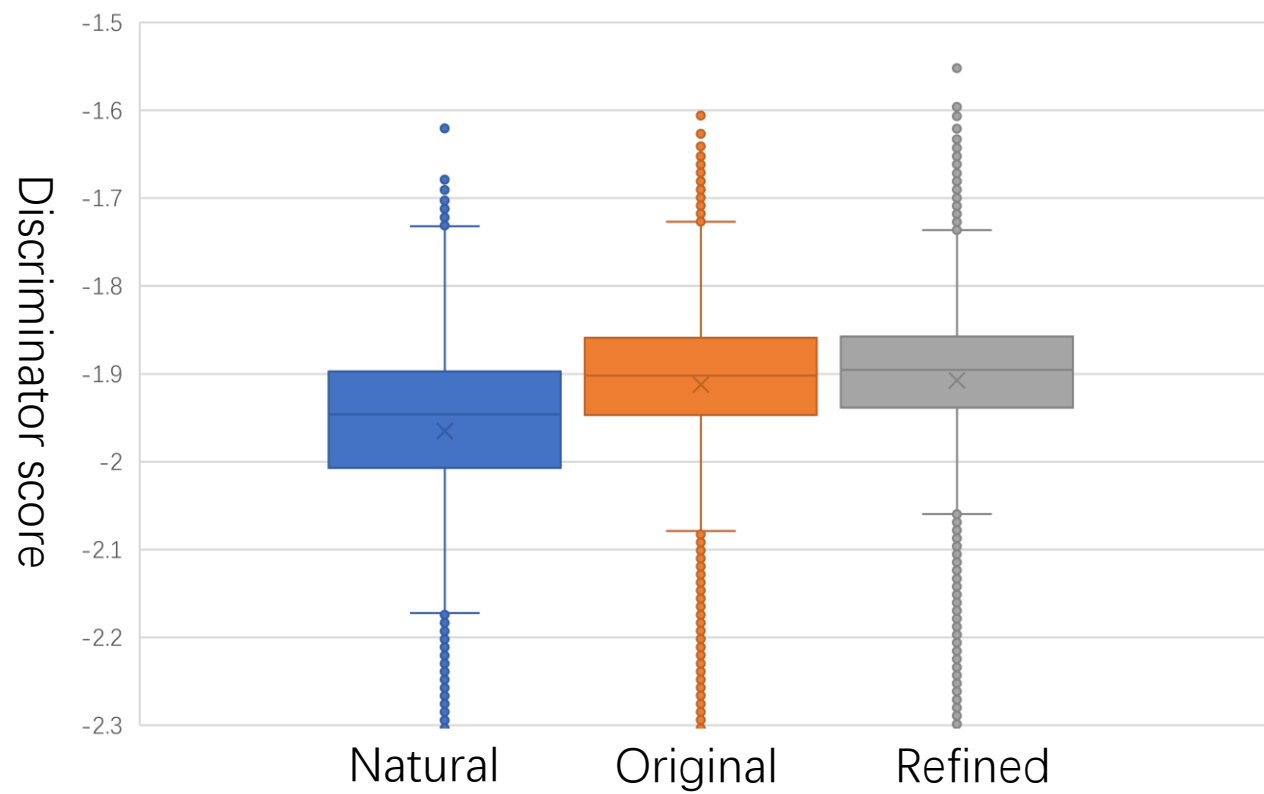

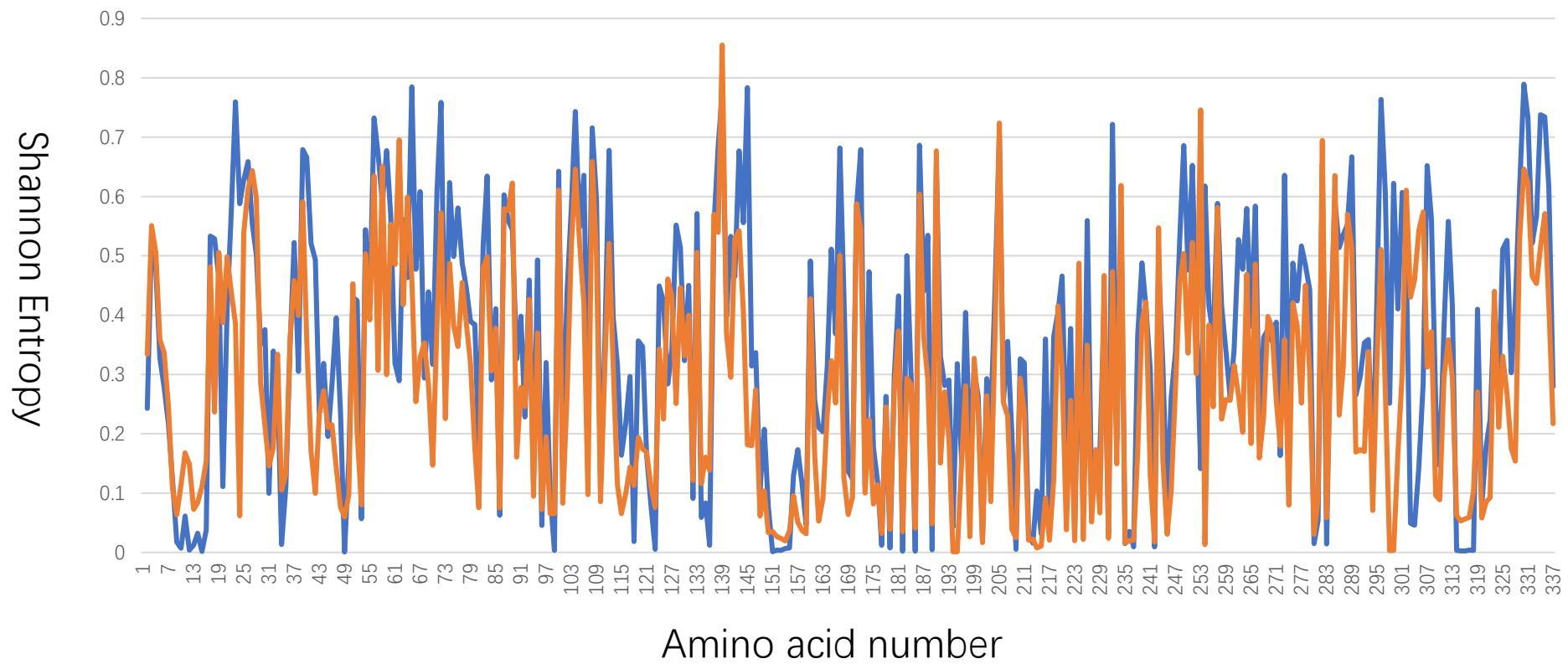

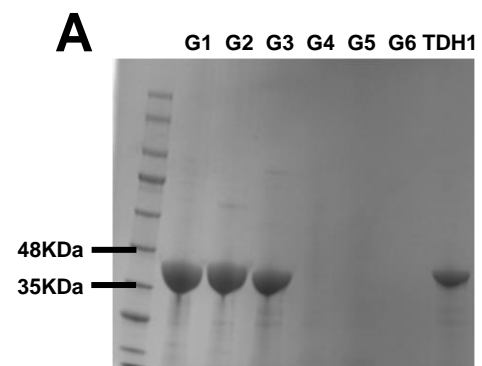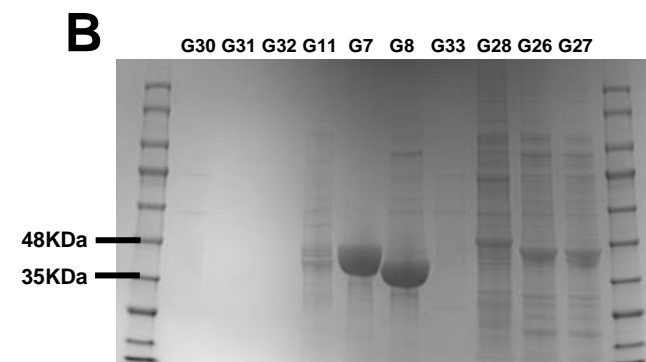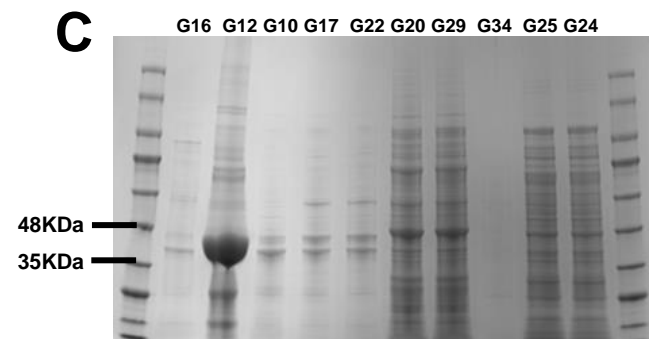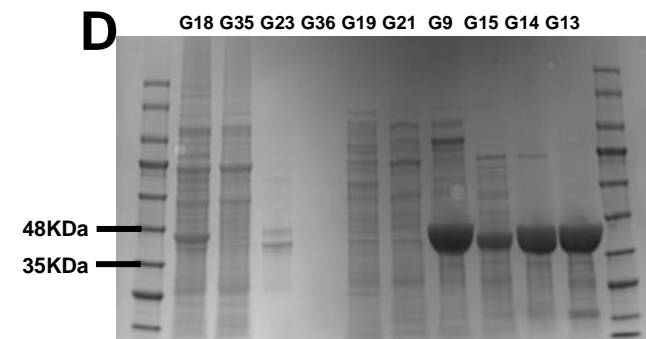

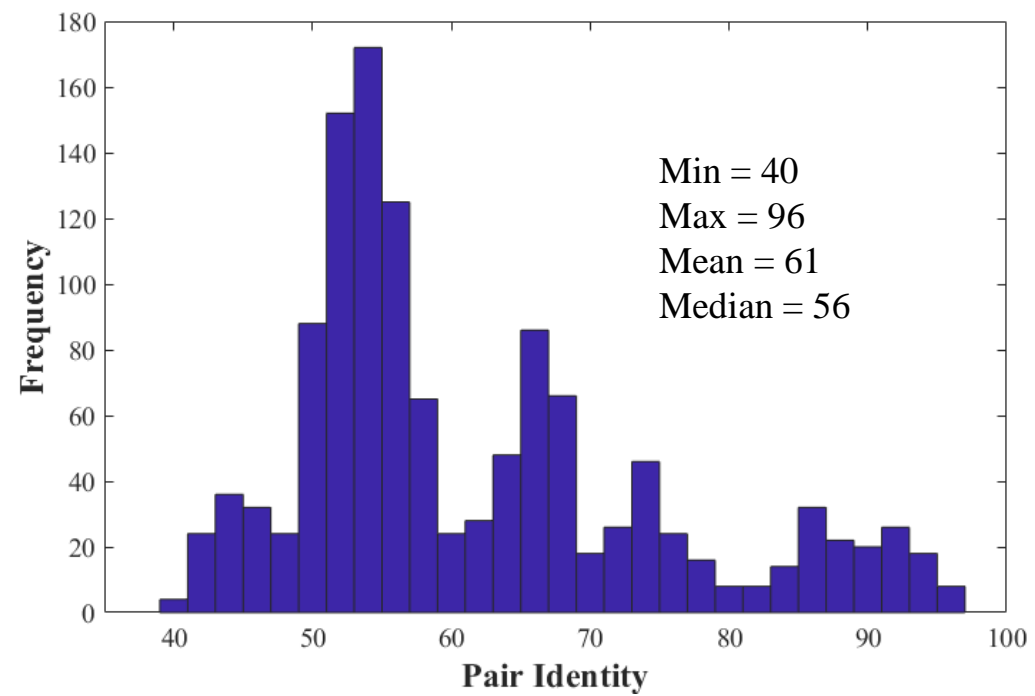

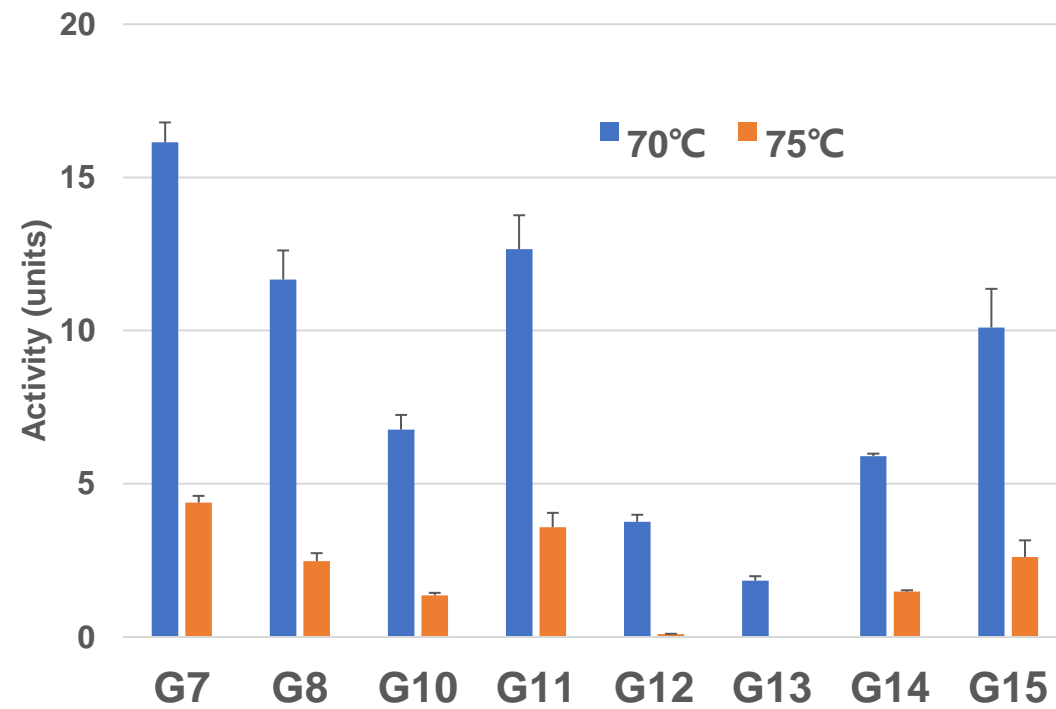

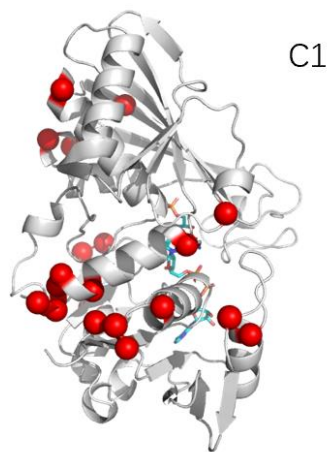

C1

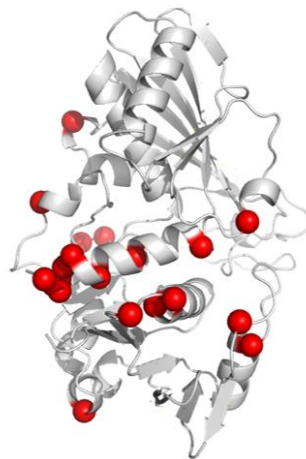

B1

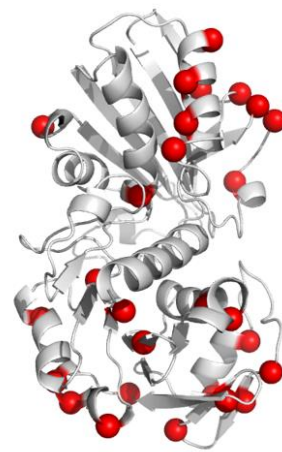

D

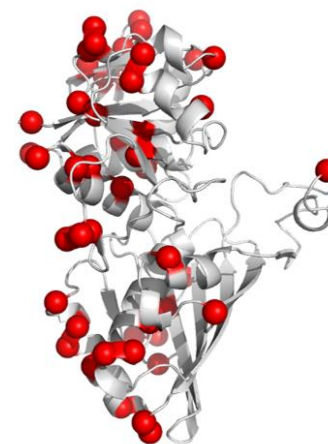

L

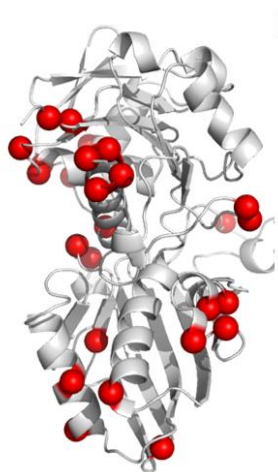

E

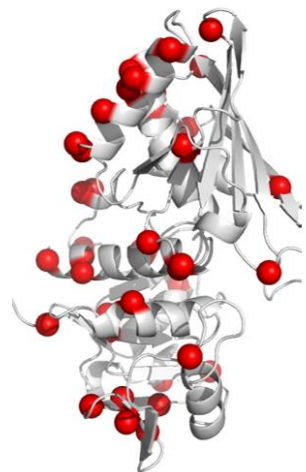

M

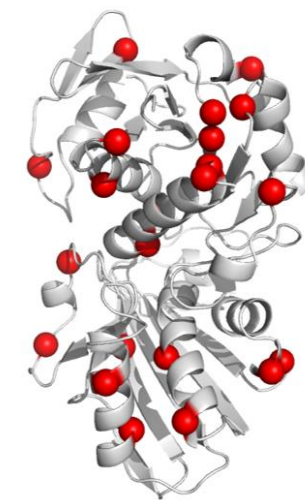

D1

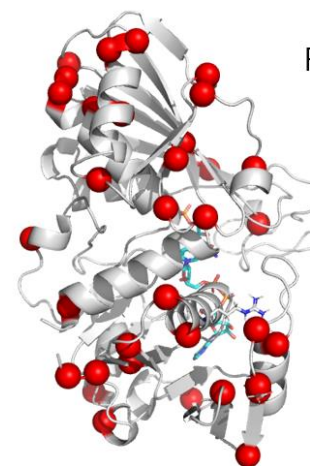

F

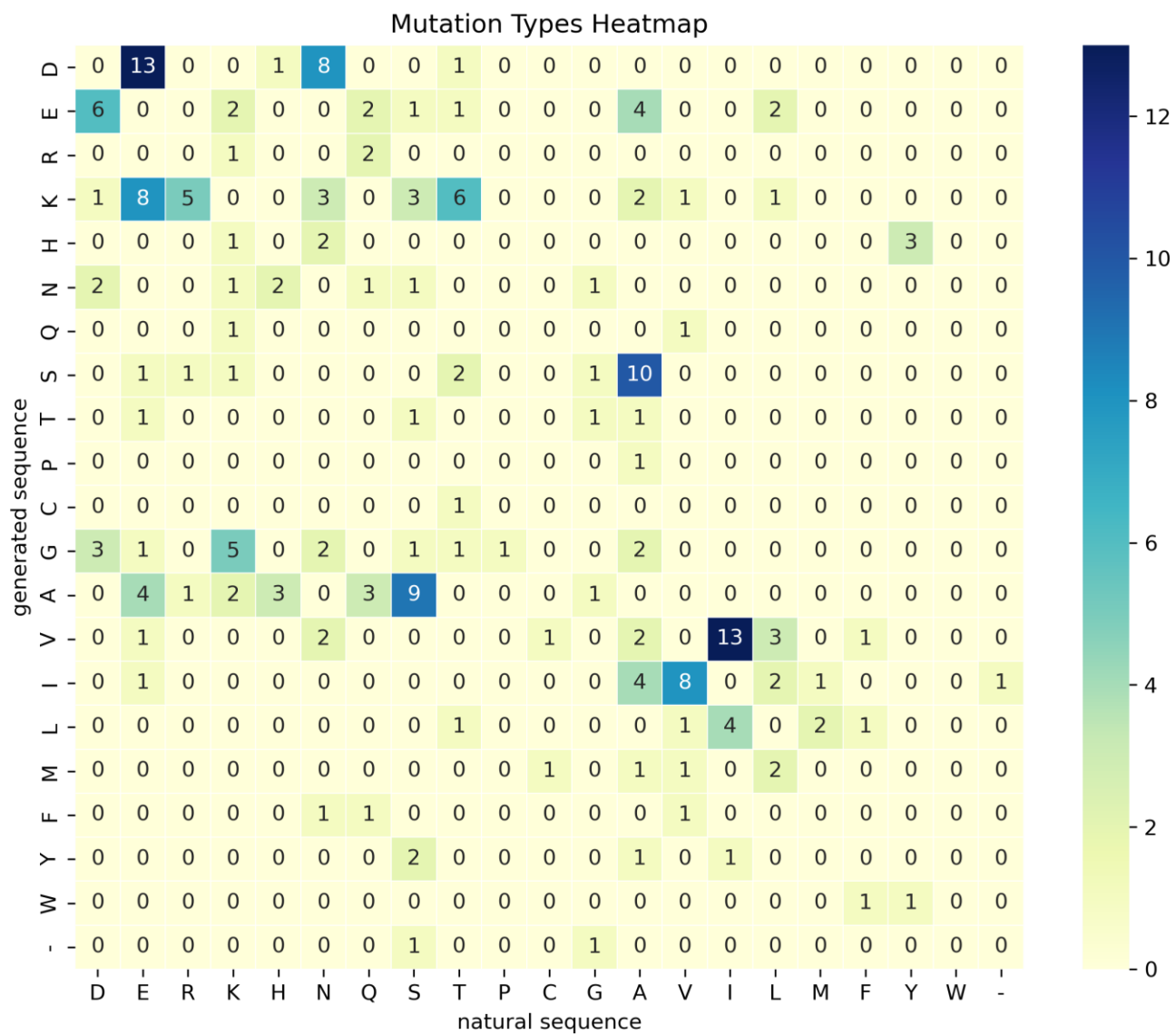

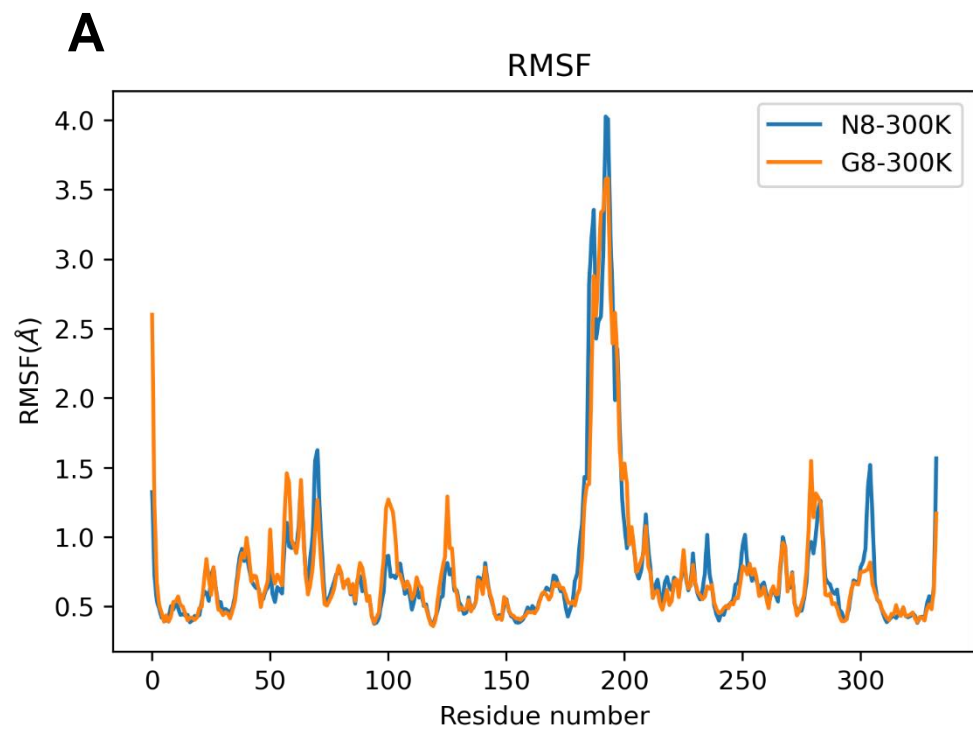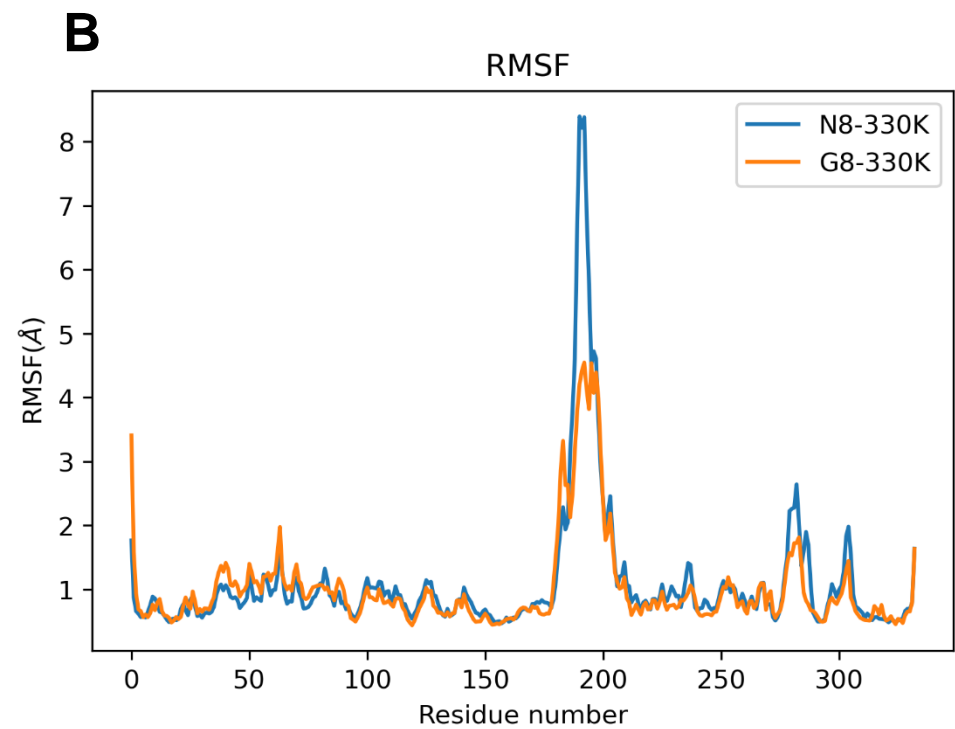

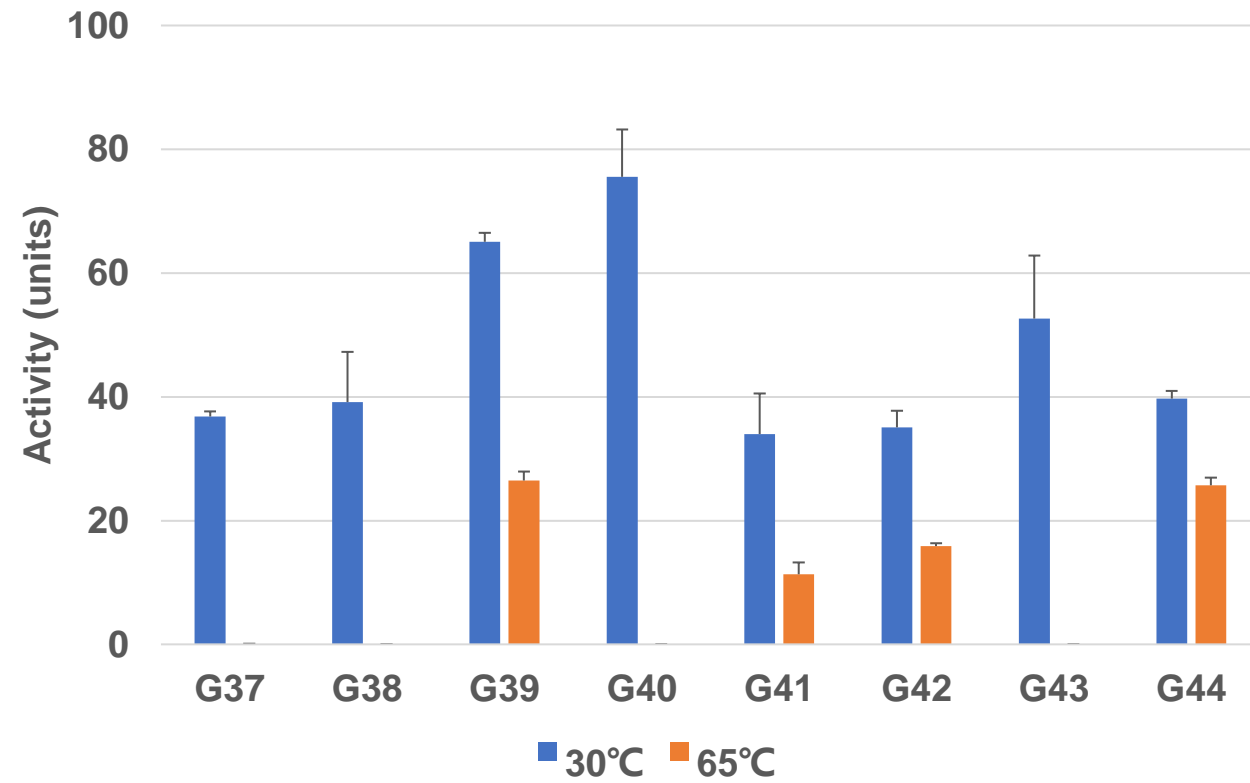

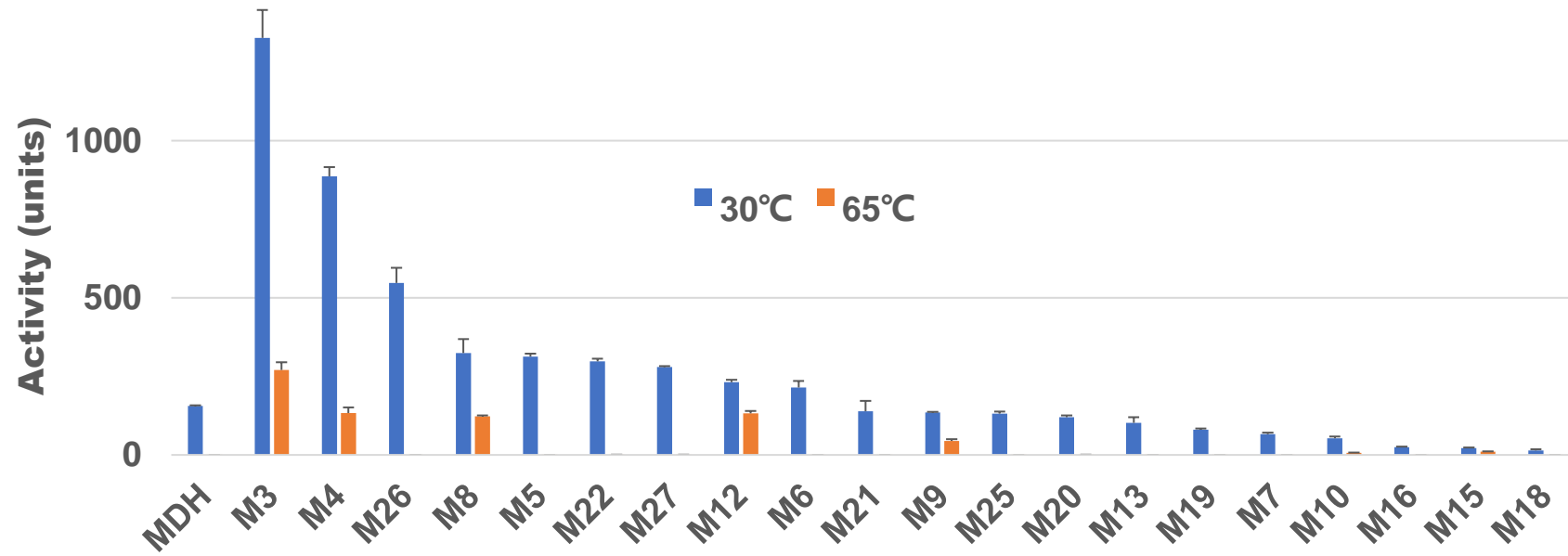

Supplement: Supplementary 1 — Figs. S1 to S15 Tables S1 to S3 [file bdr.0031.f1.zip › sup_figures.pdf]
